# Postoperative pulmonary edema following vitrectomy in patients with ischemic heart disease and diastolic dysfunction in the post-anesthetic care unit: Two case reports

**DOI:** 10.1097/MD.0000000000022296

**Published:** 2020-09-18

**Authors:** Yuseon Cheong, Namyoong Kim, Minsoo Kim, Hee-Jeong Son, Jin Huh, Seong-Sik Kang, So Young Lim, Byeongmun Hwang

**Affiliations:** a Department of Anesthesiology and Pain Medicine, Kangwon National University Hospital, School of Medicine, Kangwon National University; b Department of Anesthesiology and Pain Medicine, College of Medicine, Hallym University, Chuncheon Sacred Heart Hospital, Chuncheon, Republic of Korea.

**Keywords:** case report, diastolic dysfunction, perioperative period, pulmonary edema, vitrectomy

## Abstract

**Rationale:**

The increasing incidence of cardiac comorbidities in the elderly population has led to an increasing demand for vigilance of cardiac dysfunction induced by surgery. Favorable outcomes can be ensured in such cases by an increased awareness of cardiogenic complications, early identification of the problem, and appropriate treatment.

**Patient concerns:**

This study presents 2 cases of acute pulmonary edema (PE) that were likely caused by ischemic heart disease and diastolic dysfunction in postoperative patients, following vitrectomy, in the post-anesthetic care unit.

**Diagnoses:**

Chest x-ray and computed tomography indicated PE.

**Interventions:**

Following the diagnosis of PE, patients were intubated and transferred to the intensive care unit where 20 mg furosemide was injected and 10 μg/kg/min dobutamine was infused intravenously.

**Outcomes:**

On postoperative day 2, the patients’ vital signs were stable and there were no signs of respiratory disturbance.

**Lessons:**

Physicians should be alert to the potential development of PE as a postoperative complication in patients with left ventricular (LV) diastolic dysfunction and ischemic heart disease, even if the patient has undergone a procedure with mild hemodynamic change and minimal surgical stimulation such as vitrectomy. We propose that physicians treating elderly patients with LV diastolic dysfunction and ischemic heart disease undergoing vitrectomy should consider the use of intraoperative transthoracic echocardiogram or transesophageal echocardiogram with continuous monitoring of blood pressure, using devices such as arterial catheter devices.

## 1 Introduction

Postoperative respiratory complications are associated with high mortality and morbidity. Pulmonary edema (PE) is one of the major respiratory complications and typically has a cardiogenic etiology.^[1]^ Owing to the severity of this condition, several studies have focused on identifying the factors which predispose patients to PE. Left ventricular (LV) diastolic function has recently been reported as an independent predictor of the prognosis and adverse cardiac events, including PE, in patients undergoing cardiovascular and non-cardiovascular surgeries.^[2–4]^ Furthermore, patients with coronary artery disease exhibit high mortality rates if they develop PE.^[5]^ The increasing incidence of cardiac comorbidities in the elderly population has led to an increasing demand for vigilance of cardiac dysfunction induced by surgery. The American College of Cardiology and the American Heart Association (ACC/AHA) guidelines recommend the use of risk indices for preoperative cardiac evaluations for non-cardiac surgery.^[6]^ On the contrary, the ACC/AHA guidelines exclude perioperative cardiovascular evaluations for patients undergoing low-risk surgeries. However, perioperative cardiovascular evaluations are needed even for patients undergoing hemodynamically stable surgeries with relatively short operation times, such as vitrectomy.

We believe that favorable outcomes are ensured in such cases by anticipating the possibility of the development of cardiogenic PE, early identification of the condition should it occur, and prompt administration of appropriate treatment. Herein, we present 2 cases of acute pulmonary edema that were likely caused by ischemic heart disease and LV diastolic dysfunction in postoperative patients following vitrectomy.

## 2 Case report

### 2.1 Case 1

**Figure 1:**
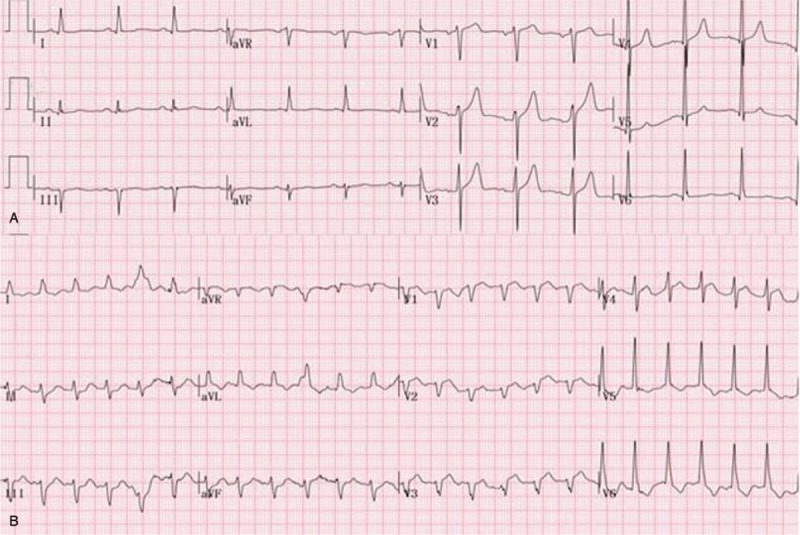
The electrocardiograph showing non-specific T wave abnormalities preoperatively (A) and ST segment depression in leads V4 to V6 postoperatively (B).

**Figure 2:**
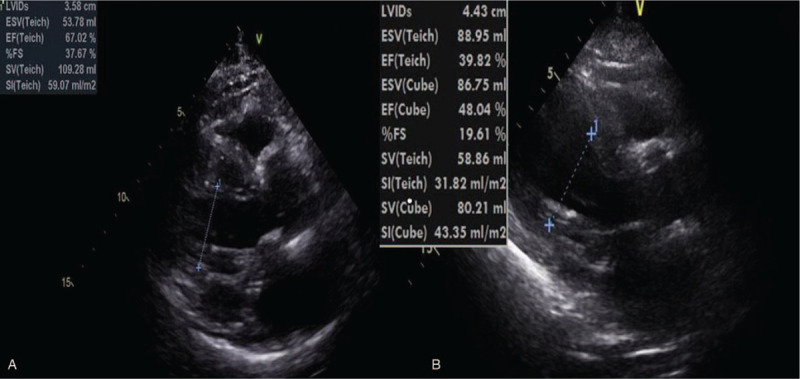
The preoperative transthoracic echocardiograph (A) showing ischemic heart disease with preserved left ventricular ejection fraction (67%) while the postoperative transthoracic echocardiograph (B) shows ischemic heart disease with moderate left ventricular systolic dysfunction (ejection fraction = 39%). EF = ejection fracture, ESV = endsystolic volume, FS = fractional shortening, LVID = Left ventricle internal diameter, SI = stroke index, SV = stroke volume.

A 77-year-old man, weighing 67 kg (body mass index: 23), was diagnosed with retinal detachment and scheduled for right total vitrectomy. He had a medical history of hypertension and non-insulin-dependent diabetes with unspecified, variant angina. He also reported a 55-pack-year smoking history. Percutaneous coronary intervention (PCI) was performed ∼10 years prior to this surgery due to acute myocardial infarction; he had also experienced an episode of acute congestive heart failure (HF) with preserved ejection fraction (EF) 2 years before this surgery. The patient had a physical status score of III according to the American Society of Anesthesiologists. During preoperative evaluation, the patient described his functional capacity as good and denied experiencing any symptoms of HF. Physical examination did not reveal any signs of congestive HF. The electrocardiograph (ECG) showed non-specific T wave abnormalities with LV hypertrophy (Fig. 1A). The transthoracic echocardiogram (TTE) revealed ischemic heart disease with normal LV size and function (EF: 67%), grade 2 diastolic dysfunction with left atrial enlargement, regional wall motion abnormalities (inferior wall hypokinesia and inferolateral wall hypokinesia), and no significant valvular pathology. In the preoperative TTE, left ventricular ejection fraction (LVEF) was 67%, the ratio of peak early to late flow of mitral inflow (E/A) was 1.02, and the ratio of LV early diastolic filling velocity to the peak diastolic velocity of the medial mitral annulus (E/e′) was 22.18 (Fig. 2A). The patient was taking clopidogrel 75 mg, atorvastatin 20 mg, and nicorandil 5 mg for treatment of cardiac disease. The preoperative cardiology consultation assessed the surgical risk of the patient to be low (<1%) given his stable control of blood pressure and a good functional capacity.

Upon the patient's arrival in the operating room, routine monitoring including noninvasive automated blood pressure, pulse oximetry, and electrocardiography was begun prior to premedication. Furthermore, the bispectral index (BIS) was monitored using VISTA (Aspect Medical Systems; MA); sensors were placed on the frontotemporal regions of the cranium, as recommended by the manufacturer, and were connected to an electroencephalogram monitor. The values of BIS were maintained between 40 and 60 during the intraoperative period. General anesthesia was induced using propofol, and tracheal intubation was performed following rocuronium-induced muscular relaxation. Anesthesia was maintained using sevoflurane (1.5–2.0 vol%) and an air–oxygen mixture (FiO_2_ 0.5). Muscle relaxation was maintained using intermittent doses of intravenous rocuronium titrated to keep the train of 4 to <2 twitches.

Blood pressure, heart rate, and peripheral oxygen saturation (SpO_2_) were 100 to 120/76 to 86 mmHg, 70 to 86 beats/min, and 99%, respectively. Blood pressure was stable at the beginning of the surgery; however, 40 minutes after anesthetic induction, it declined to a low of 80 mmHg systolic pressure. This was managed using 5 to 10 mg ephedrine and 50 μg phenylephrine, injected thrice over 30 minutes. At 30 minutes after the onset of hypotension, a radial artery catheter was placed to measure the arterial blood pressure, and a continuous intravenous infusion of 5 μg/kg/min dobutamine was initiated. Following this, the systolic blood pressure and heart rate were 100 to 120 mmHg and 80 to 100 beats/min, respectively. The patient did not have any significant electrolyte abnormalities, as determined by arterial blood gas (ABG) investigation.

**Figure 3:**
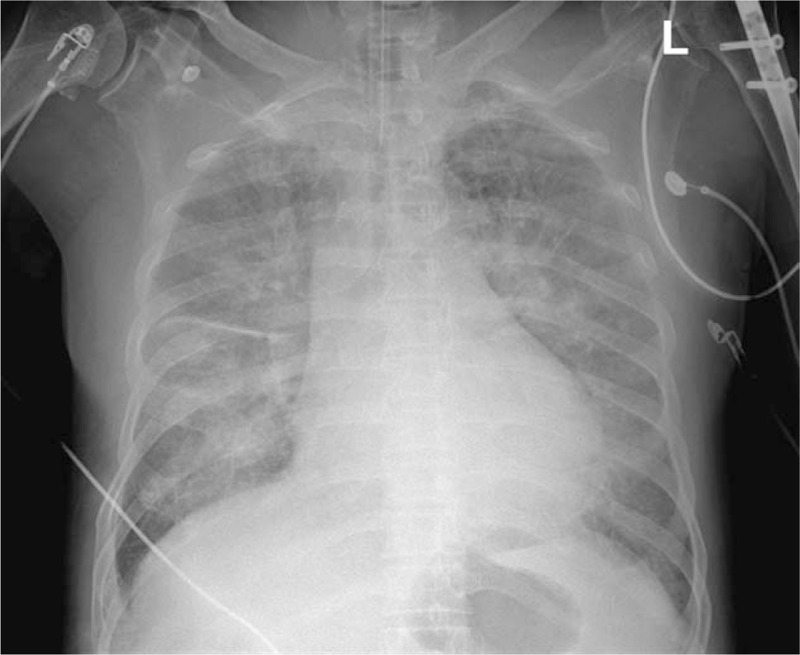
Postoperative chest x-ray showing bilateral vascular congestion.

**Figure 4:**
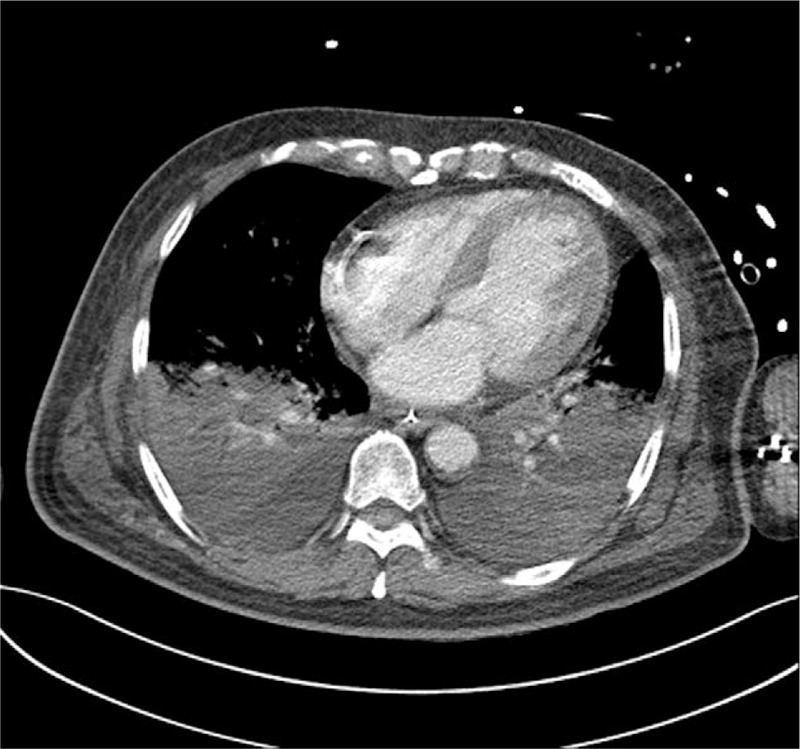
Computed tomography scans showing consolidation (bilateral pleural and fissural effusion) and interlobular septal thickening in both lungs.

The surgery lasted approximately 2 hours. The patient experienced mild bleeding and fluid replacement was performed using 300 mL of normal saline. Postoperatively, all anesthetic agents were discontinued, and the residual neuromuscular block was reversed using 15 mg pyridostigmine and 0.4 mg glycopyrrolate. The patient was responsive to verbal commands, and was extubated immediately after adequate spontaneous ventilation was ensured. He was then transferred to the post-anesthetic care unit (PACU). On arrival in the PACU, the SpO_2_ was evaluated and found to be 60%; therefore, the patient was reintubated. Suctioning of the trachea revealed pink frothy sputum. At 5 minutes after tracheal intubation, the SpO_2_ gradually increased to 90%. The patient was transferred to the surgical intensive care unit (SICU) without extubation, for further management. In the SICU, intravenous injection of 20 mg furosemide was administered, and infusion of 10 μg/kg/min dobutamine was continued. He received an additional dose of furosemide and his respiratory status improved over the next 12 hours. The immediate postoperative chest x-ray scan revealed bilateral vascular congestion (Fig. 3). The ECG showed ST depression in leads V4 to V6 (Fig. 1B). Computed tomography (CT) examination revealed multiple patchy densities throughout both lungs (Fig. 4). Postoperative TTE, revealed an EF of 39% and moderate systolic dysfunction of the mid to apical LV wall segments; diastolic dysfunction was indeterminate due to E/A summation (Fig. 2B).

On postoperative day 1, his vital signs were stable and there were no signs of respiratory disturbance. Follow-up chest radiography showed a decrease in pulmonary congestion. Therefore, extubation was performed, and diuretic (furosemide) and inotropic (dobutamine) treatment was discontinued. On postoperative day 3, he was transferred to the general ward in an overall stable condition and was discharged 5 days later without any complications. No abnormalities were observed during the follow-up period of 4 weeks.

### 2.2 Case 2

**Figure 5:**
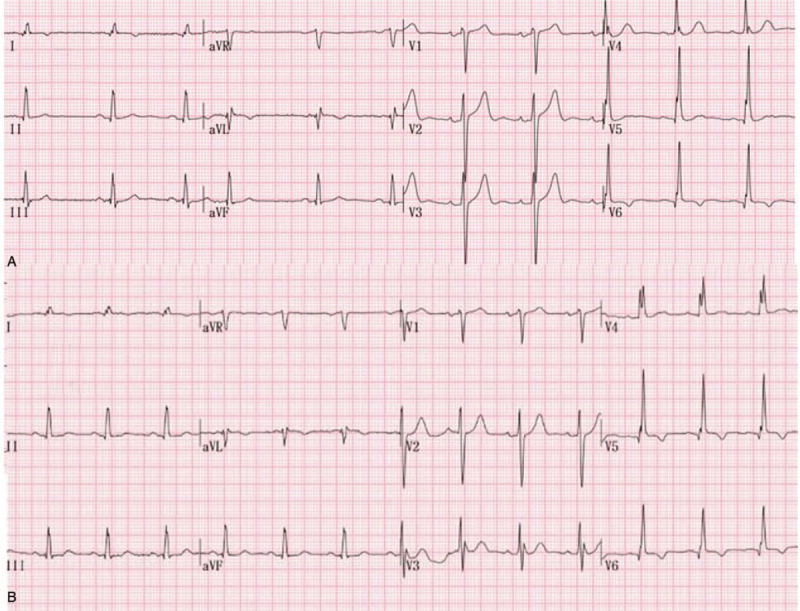
The electrocardiograph showing non-specific T wave abnormalities preoperatively (A) and ST depression in leads V5 to V6 postoperatively (B).

**Figure 6:**
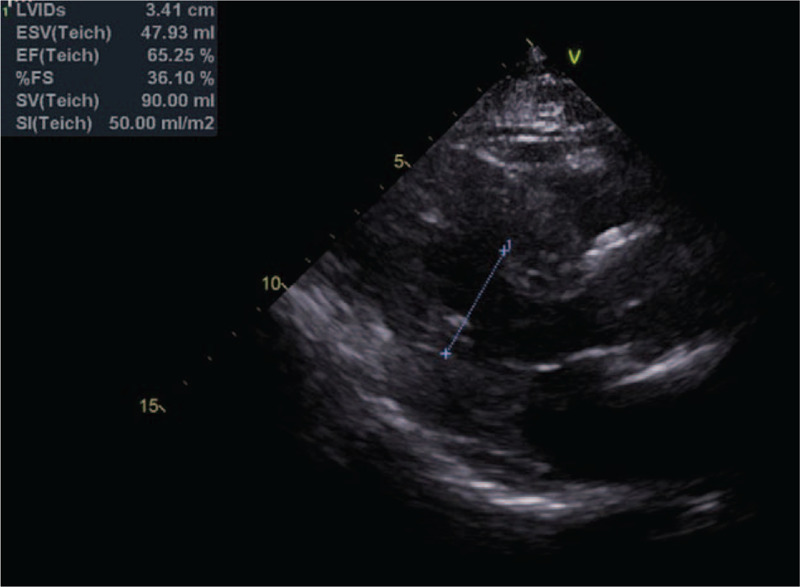
Preoperative transthoracic echocardiograph showing ischemic heart disease with preserved left ventricular ejection fraction (65%). EF = ejection fracture, ESV = endsystolic volume, FS = fractional shortening, LVID = Left ventricle internal diameter, SI = stroke index, SV = stroke volume.

A 74-year-old man, weighing 54 kg (body mass index: 21), was diagnosed with an epiretinal membrane and scheduled for left total vitrectomy. His medical history was significant for hypertension and non-insulin-dependent diabetes. There was no history of PCI or coronary angiography. The patient's physical status score was II according to the American Society of Anesthesiologists. During the preoperative evaluation, the patient described his functional capacity as good and denied experiencing any symptoms of HF. Physical examination did not reveal any signs of congestive HF. The preoperative ECG showed non-specific T wave abnormalities and atrial fibrillation (Fig. 5). TTE revealed ischemic heart disease with normal LV size and function (EF: 65%), grade 2 diastolic dysfunction with left atrial enlargement, regional wall motion abnormalities (inferior wall hypokinesia and antero-lateral wall hypokinesia), and no significant valvular pathology. Preoperative TTE revealed, LVEF = 65%, E/A = 2.64, and E/e′ = 16.43 (Fig. 6). The patient was taking spironolactone 25 mg and furosemide 40 mg for treatment of cardiac disease. The preoperative cardiology consultation estimated the surgical risk of the patient to be low (<1%) considering his blood pressure was stable and functional capacity was good.

The procedure was performed under general anesthesia and endotracheal intubation with routine monitoring as in Case 1. Anesthesia was induced using propofol, and intubation was performed following rocuronium-induced muscular relaxation. Anesthesia was maintained using sevoflurane (1.5–2.0 vol%) and an air–oxygen mixture (FiO2 0.5). Muscle relaxation was maintained using intermittent doses of intravenous rocuronium titrated to keep the train of 4 to less than 2 twitches.

**Figure 7:**
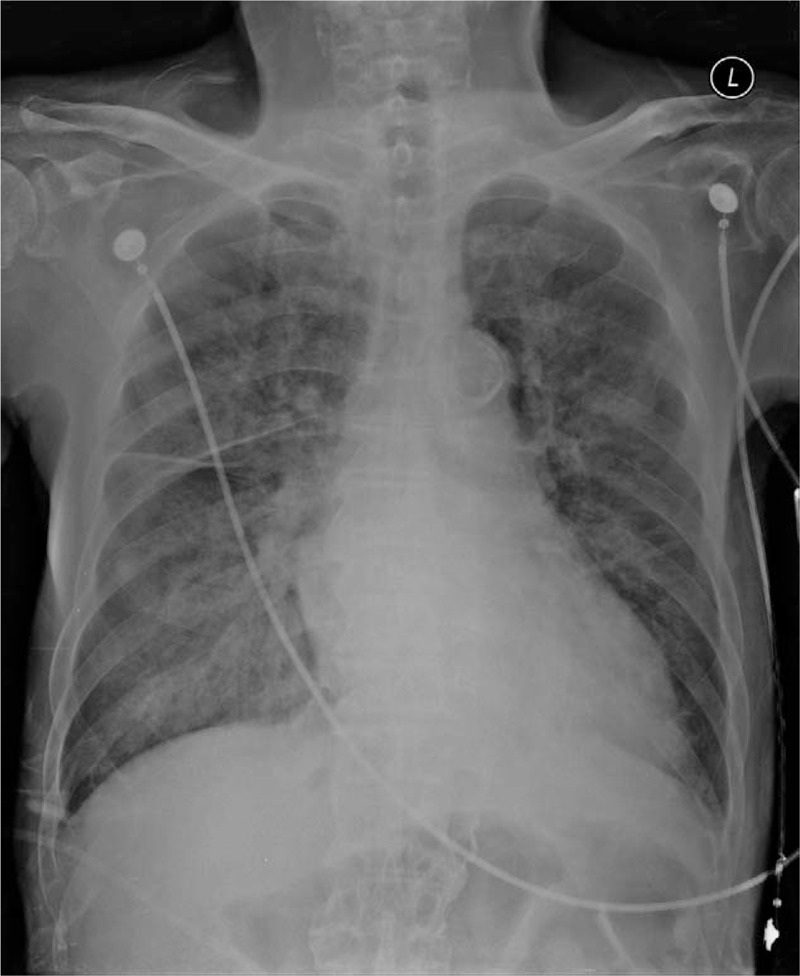
Postoperative chest x-ray showing bilateral vascular congestion.

The perioperative blood pressure was stable and the surgery lasted for approximately 80 minutes. The patient experienced mild bleeding and fluid replacement was performed with 200 mL of normal saline. Postoperatively, all anesthetic agents were discontinued and the residual neuromuscular block was reversed using 15 mg pyridostigmine and 0.4 mg glycopyrrolate. The patient was responsive to verbal commands, and was extubated immediately after adequate spontaneous ventilation was ensured. He was then transferred to the PACU. On arrival, the SpO_2_ was evaluated and found to be 99%; 30 minutes after arrival, it gradually decreased to 93%. Therefore, the patient was reintubated. Suctioning of the trachea revealed pink frothy sputum. Postoperative chest radiograph was obtained immediately (Fig. 7) and the ECG was evaluated (Fig. 5). The patient was transferred to the SICU without extubation for further management. In the SICU, 20 mg furosemide was injected intravenously, and infusion of 10 μg/kg/min dobutamine was continued. The patient received an additional dose of furosemide and his respiratory status improved over the next 12 hours. On postoperative day 1, his vital signs were stable and there were no signs of respiratory disturbance. On postoperative day 2, he was transferred to the general ward in an overall stable condition and then discharged on postoperative day 3 without any complications. No abnormalities were observed during the follow-up period of 4 weeks.

## 3 Discussion

Given the increasing incidence of cardiac comorbidities in the elderly population, there is an increasing demand for vigilance of cardiac dysfunction induced by surgery and during weaning from mechanical ventilation. In the present cases, pre-existing cardiac disease (LV diastolic dysfunction and ischemic heart disease) contributed to the occurrence of postoperative PE. Based on our study of the two cases, we believe that cardiogenic PE can occur even in patients undergoing hemodynamically stable surgeries with relatively short operation times, such as vitrectomy. Therefore, prior identification of high-risk patients is crucial for the early diagnosis of cardiogenic postoperative complications, which will, in turn, facilitate prompt and appropriate management, thereby decreasing the postoperative morbidity and mortality.

A high prevalence of diastolic dysfunction is observed in elderly patients with coronary artery disease, cardiomyopathies, valvular disease, hypertension, diabetes mellitus, and a variety of other systemic diseases.^[2,3]^ The prevalence of diastolic dysfunction in the age group of ≥60 years is estimated to be 28%.^[7]^ In these cases, the patients had hypertension, diabetes, and LV diastolic dysfunction. LV diastolic dysfunction can contribute to the development of HF during and after surgery.^[2–4]^ Preoperative LV diastolic dysfunction has recently been reported to correlate with the incidence of postoperative PE.^[4,8]^ LV end-diastolic pressure is easily elevated in the presence of LV diastolic dysfunction, which subsequently results in PE. Therefore, preoperative evaluations should include not only LV systolic function but also LV diastolic function. LV diastolic function can be assessed by echocardiography. The ratio of LV early diastolic filling velocity to the peak diastolic velocity of the medial mitral annulus (E/e′) can be used as an index of LV diastolic function. LV diastolic function is considered normal when E/e′ is <8, while LV diastolic dysfunction is defined as E/e′ >15.^[4]^ In previous studies, the E/e′ was significantly higher in patients with PE.^[8,9]^ In the cases presented here the values of E/e′ were >15 (22.18 and 16.43, respectively) for cases 1 and 2.

In a clinical setting, the shift from mechanical ventilation to spontaneous breathing may induce cardiac dysfunction and is a potential etiology underlying weaning-induced PE.^[10]^ Weaning-induced acute hemodynamic effects may have detrimental consequences in patients with cardiovascular disease, particularly in patients with coronary artery disease. These effects may also induce localized myocardial ischemia or unmask areas of pre-existing marginal function. Myocardial ischemia has been reported as a cause of PE.^[9]^ Myocardial ischemia could be the main mechanism of cardiac failure during weaning; effective management of this condition may improve the success of weaning in such cases.^[10]^ Shifts in intravascular volume, significant operative pain, or withdrawal of anesthesia may contribute to cardiac events during the weaning period.^[10]^ In the cases presented in this study, perioperative stress along with myocardial ischemia could have contributed to the development of acute PE. Furthermore, diastolic dysfunction may aggravate myocardial ischemia in addition to the stress of weaning.

Acute decompensated HF may be precipitated by myocardial ischemia.^[11,12]^ In a clinical situation such as the acute onset of severe myocardial ischemia, PE could be induced by acute decompensated HF in susceptible patients. Acute decompensated HF typically manifests as respiratory distress with PE. Any postoperative patient with suspected PE should be evaluated for new or unstable myocardial ischemia. In the present cases, the patient had ischemic heart disease and diastolic dysfunction. Therefore, in these cases, PE could be induced from acute decompensated HF with acute onset of myocardial ischemia. Even patients with no history of HF or known cases of HF who are asymptomatic can develop clinical manifestations of HF under the stress of surgery. Therefore, it is important to evaluate all patients for known risk factors of HF, especially elderly patients with other known risk factors.^[13,14]^ However, there are no guidelines for perioperative cardiovascular evaluations for patients undergoing low-risk surgeries, such as vitrectomy. Although a pre-existing cardiac disease is considered a significant risk factor for perioperative complications, there is no recommendation for perioperative monitoring in patients who are asymptomatic with LV diastolic dysfunction and ischemic heart disease. However, in light of the result of the present cases, perioperative cardiovascular evaluations and monitoring are clearly needed even for patients undergoing hemodynamically stable surgeries with relatively short operation times, such as vitrectomy.

Continuous ECG monitoring is necessary to detect myocardial ischemia. Methods of invasive monitoring, such as arterial catheter devices, in addition to standard monitoring of volume status, should be employed to avoid hypo or hypervolemia and guide fluid and diuretic administration in patients with HF caused by ischemic heart disease. We recommend that an intra-arterial catheter be inserted prior to the induction of anesthesia in all surgical patients with ischemic heart disease. Emergency intraoperative or perioperative TTE or transesophageal echocardiogram (TEE) is indicated to determine the cause of any unexplained, persistent or life-threatening hemodynamic instability. In patients at high risk for myocardial ischemia, continuous intraoperative TTE or TEE monitoring may be useful to immediately detect any new regional wall motion abnormalities suggestive of ischemia.

Excess fluid balance is also a concern with PE, especially in patients with reduced cardiac function. A continuous infusion of crystalloid may be supplemented with the administration of smaller than usual (i.e., 1–2 mL/kg/hr) crystalloid boluses in patients with decompensated HF.^[12]^ In the present cases, intravenous crystalloid fluid infusion was administrated at approximately 2.24 to 2.78 mL/kg/h during the intraoperative period. The authors did not actively perform fluid restriction since the preoperative cardiac functions were relatively good (stable control of blood pressure and good functional capacity) for both patients. There were no significant differences in the fluid in-out balance during the perioperative period. We believe that it is unlikely that the fluid administration contributed to the initial episode of acute PE as there were no significant fluid shifts observed in our patients.

During the intraoperative period, there was an episode of hypotension. At the time, we assumed that this hypotension was temporary. Therefore, we did not perform aggressive treatment or monitoring. Consequently, we now believe that aggressive treatment might have been necessary, considering that temporary hypotension during surgery could have been caused by cardiac depression. Moreover, invasive measurement of arterial blood pressure and the use of TEE in the intraoperative period may have been necessary to prevent the occurrence of PE. In the cases presented here, we believe that PE occurred due to the worsening of cardiac function during the weaning process with cardiac depression during surgery. Conversely, there is the possibility that PE might have occurred during the intraoperative period. Although our patient showed no decrease in SpO_2_, frequent hypotension could imply the occurrence of PE during the intraoperative period.

When PE was diagnosed, we used an inotropic agent, 10 μg/min/kg dobutamine, based on the conclusion that the patient had severe systolic dysfunction and low cardiac output syndrome. We also used a diuretic agent, 20 mg/12 h furosemide, due to the diagnosis of PE. Management of patients with PE due to diastolic LV dysfunction should include the maintenance of adequate preload (including fluid removal if necessary), slower heart rate to allow an adequate diastolic filling time, and the avoidance of hypertension to decrease the afterload to the left ventricle.^[1,12]^

An alternative cause of PE in the present cases may have been negative pressure PE. However, this pathogenesis is contradicted in the present cases because there were no intracranial lesions or upper airway obstructions in the perioperative period.^[15]^ Furthermore, the patients discussed in this case study were elderly, which is not supportive of the diagnosis of negative pressure PE, which mainly occurs in relatively younger patients.

A further prospective study for adequate perioperative monitoring and management in patients with LV diastolic dysfunction and ischemic heart disease undergoing low-risk surgeries is warranted to decrease perioperative complication, morbidity, and mortality.

## 4 Conclusions

Physicians should be alert to the potential development of postoperative PE in patients with LV diastolic dysfunction and ischemic heart disease, even if the patient has undergone surgeries with only mild hemodynamic changes and minimal surgical stimulation such as vitrectomy. Therefore, heightened vigilance of cardiac dysfunction is essential to prevent this perioperative complication. We propose that physicians treating elderly patients with LV diastolic dysfunction and ischemic heart disease undergoing vitrectomy should consider the use of intraoperative transthoracic echocardiogram or transesophageal echocardiogram with continuous monitoring of blood pressure, using devices such as arterial catheter devices.

## Author contributions

**Conceptualizations:** Yuseon Cheong, Byeongmun Hwang.

**Data curation:** Namyoong Kim, Minsoo Kim.

**Formal analysis:** Hee-Jeong Son, So Young Lim, Seong-Sik Kang, Jin Huh.

**Investigation:** Byeongmun Hwang.

**Writing – original draft:** Minsoo Kim, Byeongmun Hwang.

**Writing – review &editing:** Byeongmun Hwang.

## Abbreviations

Abbreviations: LV = left ventricle, PE = pulmonary edema.
